# Rapid loss of group 1 innate lymphoid cells during blood stage *Plasmodium* infection‡

**DOI:** 10.1002/cti2.1003

**Published:** 2018-01-12

**Authors:** Susanna S Ng, Fernando Souza‐Fonseca‐Guimaraes, Fabian de Labastida Rivera, Fiona H Amante, Rajiv Kumar, Yulong Gao, Meru Sheel, Lynette Beattie, Marcela Montes de Oca, Camille Guillerey, Chelsea L Edwards, Rebecca J Faleiro, Teija Frame, Patrick T Bunn, Eric Vivier, Dale I Godfrey, Daniel G Pellicci, J Alejandro Lopez, Katherine T Andrews, Nicholas D Huntington, Mark J Smyth, James McCarthy, Christian R Engwerda

**Affiliations:** ^1^ Immunology and Infection Laboratory QIMR Berghofer Medical Research Institute Herston QLD Australia; ^2^ School of Natural Sciences Griffith University Nathan QLD Australia; ^3^ Faculty of Medicine, Dentistry and Health Sciences University of Melbourne Melbourne VIC Australia; ^4^ Molecular Immunology Division The Walter and Eliza Hall Institute of Medical Research Parkville VIC Australia; ^5^ Department of Biochemistry Banaras Hindu University Varanasi India; ^6^ Immunology in Cancer and Infection QIMR Berghofer Medical Research Institute Herston QLD Australia; ^7^ School of Medicine University of Queensland Herston QLD Australia; ^8^ National Centre for Immunisation Research and Surveillance Westmead NSW Australia; ^9^ Aix Marseille Université, CNRS, INSERM, CIML Marseille France; ^10^ Service d'Immunologie APHM, Hôpital de la Conception Marseille France; ^11^ Department of Microbiology and Immunology Peter Doherty Institute for Infection and Immunity University of Melbourne Melbourne VIC Australia; ^12^ Australian Research Council Centre of Excellence for Advanced Molecular Imaging University of Melbourne Melbourne VIC Australia; ^13^ Department of Medical Biology The University of Melbourne Melbourne VIC Australia; ^14^ Clinical Tropical Medicine QIMR Berghofer Medical Research Institute Herston QLD Australia

**Keywords:** inflammation, natural killer cells, parasitic–protozoan

## Abstract

**Objectives:**

Innate lymphoid cells (ILCs) share many characteristics with CD4^+^ T cells, and group 1 ILCs share a requirement for T‐bet and the ability to produce IFNγ with T helper 1 (Th1) cells. Given this similarity, and the importance of Th1 cells for protection against intracellular protozoan parasites, we aimed to characterise the role of group 1 ILCs during *Plasmodium* infection.

**Methods:**

We quantified group 1 ILCs in peripheral blood collected from subjects infected with with *Plasmodium falciparum* 3D7 as part of a controlled human malaria infection study, and in the liver and spleens of *Pc* AS‐infected mice. We used genetically‐modified mouse models, as well as cell‐depletion methods in mice to characterise the role of group 1 ILCs during *Pc* AS infection.

**Results:**

In a controlled human malaria infection study, we found that the frequencies of circulating ILC1s and NK cells decreased as infection progressed but recovered after volunteers were treated with antiparasitic drug. A similar observation was made for liver and splenic ILC1s in *P. chabaudi chabaudi* AS (*Pc* AS)‐infected mice. The decrease in mouse liver ILC1 frequencies was associated with increased apoptosis. We also identified a population of cells within the liver and spleen that expressed both ILC1 and NK cell markers, indicative of plasticity between these two cell lineages. Studies using genetic and cell‐depletion approaches indicated that group 1 ILCs have a limited role in antiparasitic immunity during *Pc* AS infection in mice.

**Discussion:**

Our results are consistent with a previous study indicating a limited role for natural killer (NK) cells during *Plasmodium chabaudi* infection in mice. Additionally, a recent study reported the redundancy of ILCs in humans with competent B and T cells. Nonetheless, our results do not rule out a role for group 1 ILCs in human malaria in endemic settings given that blood stage infection was initiated intravenously in our experimental models, and thus bypassed the liver stage of infection, which may influence the immune response during the blood stage.

**Conclusion:**

Our results show that ILC1s are lost early during mouse and human malaria, and this observation may help to explain the limited role for these cells in controlling blood stage infection.

## Introduction

Innate lymphoid cells (ILCs) resemble T helper (Th) cells in terms of their characteristic transcription factors and functions.^1^ Groups 1, 2 and 3 ILCs make up the ILC repertoire, and these groups are similar to Th1, Th2 and Th17 cells, respectively. In contrast to Th cells of the adaptive immune system, ILCs do not express antigen‐specific T‐cell receptors.^2^ ILCs have both protective and pathogenic roles in infectious and inflammatory diseases.^3^, ^4^ However, a recent study has suggested that ILCs are redundant in the presence of a competent B and T‐cell response in humans.^5^

Group 1 ILCs consist of conventional natural killer (cNK) cells and ILC1s.^1^, ^6^ These cell subsets share common developmental requirements for the transcription factor T‐bet and their ability to produce the pro‐inflammatory cytokine IFNγ.^6^ However, the relationship between these cells is still widely debated. Liver ILC1s are a specialised subset of group 1 ILCs also known as tissue‐resident NK (trNK) cells.^7^ Other ILC1s have also been identified in the uterus,^8^ spleen,^9^ salivary gland,^10^ kidney,^11^ adipose tissue^12^ and gastrointestinal tract.^9^ These different tissue‐resident subsets have unique cell surface phenotypes and functions.^4^, ^9^, ^13^

Malaria is a globally important infectious disease caused by protozoan parasites belonging to the genus *Plasmodium*. Following infection, a Th1‐dependent immune response can develop in the mammalian host, aiding clearance of parasites via IFNγ‐dependent mechanisms.^14^, ^15^ IFNγ production by antigen‐specific CD4^+^ T cells during *P. chabaudi chabaudi* AS (*Pc*AS) infection in mice has been reported,^16^ and increased parasite growth was observed following IFNγ neutralisation.^17^ The relationship between IFNγ production and control of parasite growth has also been reported in humans during blood stage *Plasmodium falciparum* (*Pf*) infection.^18^ Additionally, we recently showed an inverse correlation between IFNγ levels and parasite burden during controlled human malaria infection (CHMI) with *Pf*.^19^ However, the secretion of IFNγ contributes to an inflammatory environment that can also contribute to pathology.^18^, ^20^, ^21^, ^22^, ^23^, ^24^ While the adaptive immune response generated in response to *Plasmodium* infection has been well characterised, less is known about the innate immune response following infection. Early studies revealed that the depletion of NK cells with anti‐asialo GM1 antibody resulted in increased parasitaemia during *Pc*AS infection.^25^ However, the effects of this treatment on dendritic cell (DC) function^26^ and the depletion of other cell subsets, such as basophils,^27^ may impact the interpretation of these results. In fact, depletion of NK cells in mice via administration of anti‐NK1.1 antibody resulted in no effect on the course of *P. chabaudi adami* 556KA infection.^28^ However, evidence for direct interactions between human NK cells and *Pf* parasitised red blood cells (pRBC) *in vitro*, which stimulates production of IFNγ, has been reported.^29^

Given that group 1 ILCs function like Th1 cells, and little is known about their roles during *Plasmodium* infection, we examined these cells, as well as the more well‐studied innate‐like T cells (including γδ T cells,^28^ invariant natural killer T (iNKT) cells^30^, ^31^ and mucosal‐associated invariant T (MAIT) cells^32^) in volunteers infected with *Pf* in CHMI studies. Concurrently, we also investigated the role of ILC1s in C57BL/6J mice infected with *Pc*AS, which causes a chronic, nonlethal infection.^33^ We report that cNK cells and ILC1s had a limited role in controlling peripheral blood parasitaemia in mice infected with *Pc*AS. However, we found a loss in circulating NK cells and ILC1s in volunteers participating in CHMI studies with *Pf*, which was independent of parasite burden. A similar loss of ILC1s was also observed in the liver and spleen of mice infected with *Pc*AS. Furthermore, we report a novel NK1.1^+^ NKp46^+^ population that expressed both the ILC1 marker CD49a and the cNK marker DX5 (CD49b) in the livers of *Pc*AS‐infected mice, suggesting plasticity between these cell populations.

## Results

### The frequency and number of ILC1s declined during blood stage *Pf* infection

**Figure 1:**
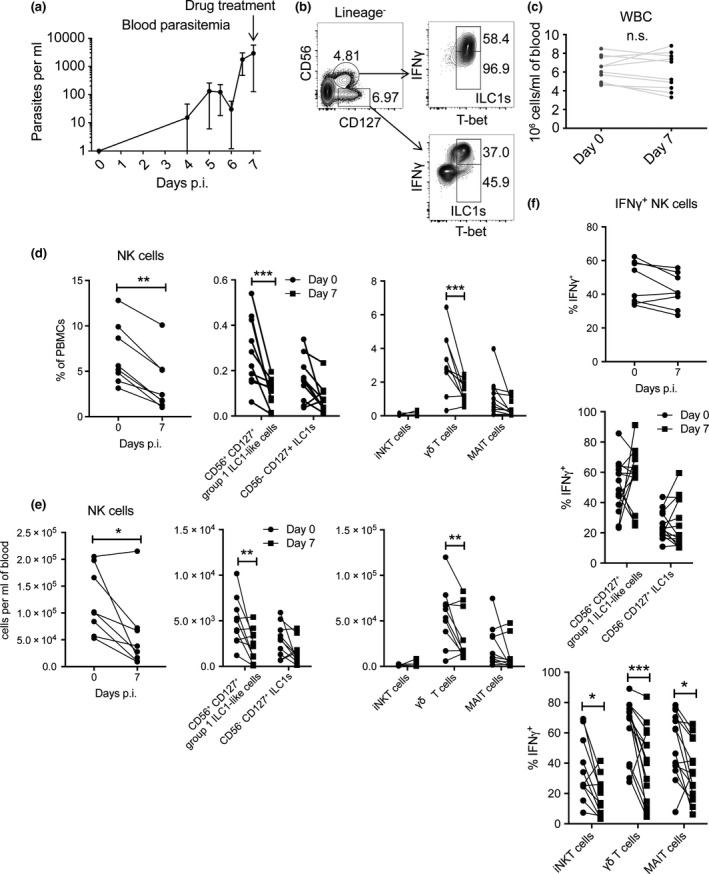
ILC and innate‐like T‐cell frequencies decrease following *P. falciparum* infection. Representative blood parasitaemia curve over the first 7 days of infection from a single cohort (*n* = 6) **(a)**. Group 1 ILC and group 1 ILC‐like subsets were identified by flow cytometry as indicated in the gating strategy **(b)**. White blood cell counts for each patient at days 0 and 7 are depicted **(c)**. The frequencies **(d)** and cell numbers **(e)** of group 1 ILC, group 1 ILC‐like and innate‐like T‐cell subsets are shown. The proportion of each subset producing IFNγ is shown **(f)**. The data from b–f represent results from one to three cohorts (*n* = 8–14). Error bars represent the mean ± standard deviation (SD) **(a)**. Comparisons between days 0 and 7 were made using the Wilcoxon (paired, nonparametric) test for NK cells and a two‐way ANOVA with Sidak's multiple comparisons test for other subsets. *P <* *0.05, **P <* *0.01, ***P <* *0.001.

NK and γδ T cells produce IFNγ in response to *Pf* infection.^34^, ^35^, ^36^ To gain a better understanding of IFNγ production by innate immune cells, including more recently identified ILC1s and innate‐like T cells, we examined these cell populations during an experimentally induced blood stage malaria infection in healthy volunteers with no prior exposure to malaria or residence in malaria‐endemic regions.^37^, ^38^ Human PBMCs were isolated from blood drawn prior to infection (day 0) and at 7 days postinfection (p.i.), prior to drug treatment (Figure 1a). We then identified group 1 ILCs (CD56^−^ CD127^+^ T‐bet^+^ ILC1s and NK cells), group 1 ILC‐like cells (CD56^+^ CD127^+^ T‐bet^+^) (Figure 1b and Supplementary figure 1A), as well as innate‐like T cells (γδ T cells [CD3^+^, γδ TCR^+^], iNKT cells [CD3^+^, CD1d PBS44 tetramer^+^] and MAIT cells [CD3^+^, CD8^+^, CD161^+^, TCR Vα7.2^+^]) (Supplementary figure 1B).

We found the frequency and number of NK cells, CD56^+^ CD127^+^ ILC1s and γδ T cells were reduced at 7 days p.i., compared to day 0 (Figures 1d and e). NK cells and all innate‐like T‐cell populations produced IFNγ upon PMA + ionomycin restimulation; however, only the frequency of IFNγ^+^ iNKT, γδ T and MAIT cells was significantly reduced at day 7, compared to day 0 (Figure 1f). The white blood cell count (WBC) in volunteers was not significantly different between days 0 and 7 p.i. (Figure 1c), indicating that a general loss of blood leukocytes during infection did not account for these results. When the frequency and numbers of these cells were examined in one cohort after volunteers were treated with antiparasitic drug, we found they had recovered and in some cases were increased, relative to pre‐infection levels (Table 1).

**Table 1:** Frequencies and total cell numbers of innate lymphoid cells and innate‐like T cells

Cell population	% of PBMCs		Cell number	
	Naive	D14	Naive	D14
CD56^‐^ CD127^+^ ILC1s	0.201 ± 0.043^a^	7.657 ± 1.473^b^	3458 ± 803	113 068 ± 21 815^b^
CD56^+^ CD127^+^ ILC1s	0.351 ± 0.060	0.497 ± 0.100	5955 ± 1084	7699 ± 1786
ILC2s	0.005 ± 0.001	0.024 ± 0.006^b^	88 ± 20	348 ± 83^b^
ILC3s	0.075 ± 0.012	0.147 ± 0.019^b^	1311 ± 263	2226 ± 326
NK cells	8.555 ± 1.922	10.073 ± 3.126	144 535 ± 29 365	144 678 ± 44 178
iNKT cells	0.071 ± 0.019	0.040 ± 0.006	1262 ± 374	587 ± 88^b^
γδ T cells	3.042 ± 0.629	4.957 ± 0.898^b^	50 915 ± 10 533	77 968 ± 14 438^b^
MAIT cells	1.245 ± 0.578	1.514 ± 0.747	22 785 ± 11 294	23 625 ± 11 166
IFNγ^+^ CD56^−^ CD127^+^ ILC1s	0.147 ± 0.037	0.374 ± 0.069^b^	2521 ± 640	5670 ± 1142
IFNγ^+^ CD56^+^ CD127^+^ ILC1s	0.162 ± 0.038	0.390 ± 0.095	2762 ± 592	6024 ± 1661
IL‐13^+^ ILC2s	0.002 ± 0.001	0.004 ± 0.001	42 ± 14	59 ± 13
IL‐22^+^ ILC3s	0.007 ± 0.002	0.017 ± 0.005	103 ± 22	238 ± 53
IFNγ^+^ NK cells	3.670 ± 1.041	4.763 ± 1.585	61 618 ± 16 064	67 648 ± 22 301
IFNγ^+^ iNKT cells	0.017 ± 0.005	0.008 ± 0.001	313 ± 119	124 ± 17
IFNγ^+^ γδ T cells	1.664 ± 0.413	3.338 ± 0.667^b^	28 636 ± 7363	52 038 ± 10 180^b^
IFNγ^+^ MAIT cells	0.643 ± 0.379	0.684 ± 0.309	12 025 ± 7268	10 625 ± 4641

a Mean ± standard error of mean (SEM) of *n* = 6 from one cohort treated with artefenomel (OZ439) on day 7 (D7) post‐infection.

b *P* value < 0.05.

Comparisons between days 0 (naive) and 14 (D14) were made using the Wilcoxon (paired, nonparametric) test.

Parasite accumulation in volunteers, as measured by the area under the curve (AUC) of blood parasitaemia curves (Figure 1a), was plotted against the frequency or cell number of each cell subset shown in Figure 1 at day 7 p.i. to identify any relationships with parasite burden. However, no significant relationships were found for any ILC or innate‐like T cells (*P* > 0.05 for all cell subsets; data not shown). Similar results were obtained when the parasite multiplication rate (PMR) over time in each volunteer was plotted against corresponding ILC or innate‐like T‐cell frequencies or cell number. Together, these results show the frequency and cell number of group 1 ILCs were reduced following first exposure to blood stage *Pf* but this reduction was independent of parasite burden or PMR and recovered following antiparasitic drug treatment. These data suggest that NK cells and ILC1s either have increased cell death, decreased cell proliferation or sequester to tissues following *Pf* infection.

### A loss of liver trNK cells and splenic ILC1s during *Pc*AS infection

**Figure 2:**
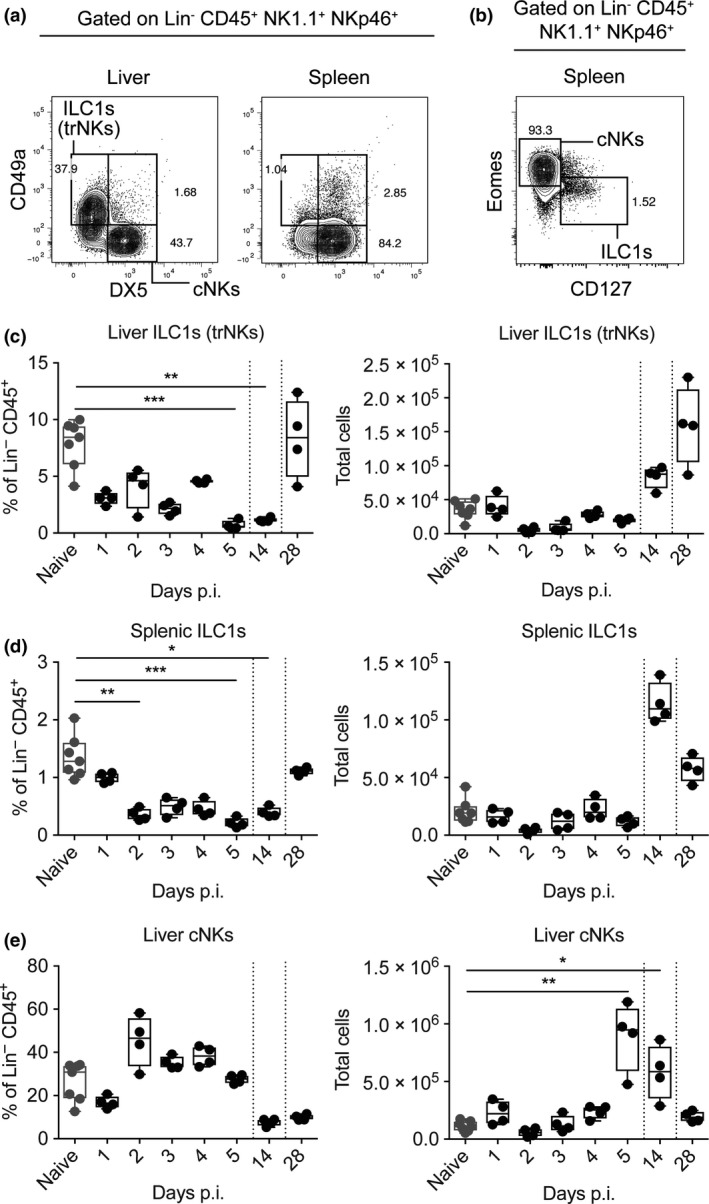
ILC1s decrease in number and frequency 5 days postinfection with *Pc*AS. The gating strategy to identify liver ILC1s, a population that is absent in the spleen is shown **(a)**, as well as a summary of the gating strategy for splenic ILC1s **(b)**. These are representative plots from naïve animals. Liver and spleen single‐cell suspensions in naïve and *Pc*AS‐infected mice were stained to determine ILC1 frequencies and cell numbers at days 1–5, 14 and 28 postinfection. Liver ILC1 **(c)**, splenic ILC1 **(d)** and liver cNK **(e)** frequencies and absolute numbers are shown. Data are representative of two experiments from cohorts of at least *n* = 4 mice per time point. Comparisons were made using the Kruskal–Wallis test accompanied by the Dunn's multiple comparisons test. *P <* *0.05, **P <* *0.01.

To further investigate ILCs in tissues during malaria, we next employed a mouse model of *PcAS* infection. A novel subset of liver ILC1s (trNK cells) has been reported in mice and humans.^7^, ^39^ We examined these cells, as well as splenic ILC1s,^9^ because of the importance of the liver and spleen as blood filtering organs during *Plasmodium* infection.^40^, ^41^ We identified liver ILC1s that were lineage (Lin; CD3, CD5, CD19)‐negative, CD45^+^ NK1.1^+^ NKp46^+^ CD49a^+^ DX5^−^ (Figure 2a). These were distinct from splenic ILC1s, identified as Lin^−^ CD45^+^ NK1.1^+^ NKp46^+^ Eomes^−^ CD127^+^ 9 (Figure 2b). We found a decrease in the frequency and number of liver (Figure 2c) and spleen ILC1s (Figure 2d) 5 days p.i. with *Pc*AS, although statistical differences were only reached in cell frequencies (Figures 2c and d). In contrast, liver cNK cells (NK1.1^+^ NKp46^+^ CD49a^−^ DX5^+^) increased in cell number over this same time period (Figure 2e).

### ILC1s exhibit a more apoptotic phenotype than cNK cells

**Figure 3:**
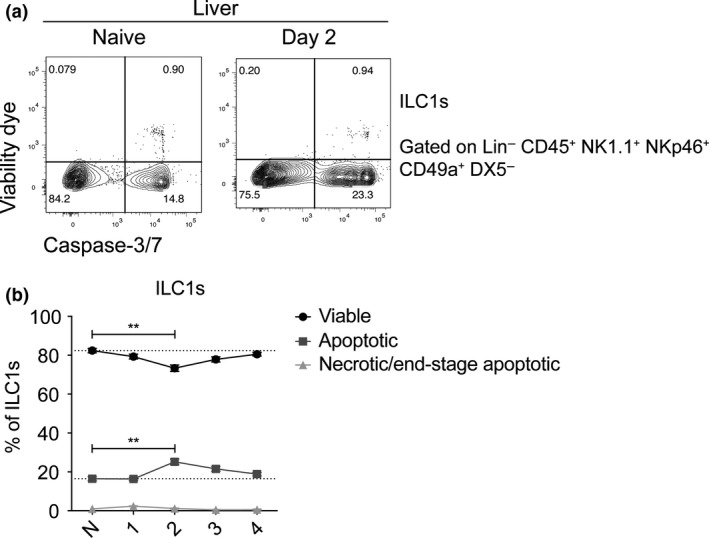
Liver ILC1s exhibit a more apoptotic phenotype than cNK cells. Liver single‐cell suspensions were stained for a viability marker, ILC1 surface markers, followed by staining for Caspase‐3/7. The representative plots from naïve and day 2 p.i. are shown **(a)** where viability dye^+^ Caspase‐3/7^−^ = dead cells, viability dye^+^ Caspase‐3/7^+^ = necrotic cells, **(b)** viability dye^−^ Caspase‐3/7^+^ = apoptotic cells, and viability dye^−^ Caspase‐3/7^−^ = live cells. The relative frequencies of viable, apoptotic and necrotic/end‐stage apoptotic ILC1s are shown. Data represent mean ± SEM from one experiment where *n* = 3 for naïve mice and *n* = 4 for *Pc*AS‐infected mice. Comparisons were made using the Kruskal–Wallis test accompanied by the Dunn's multiple comparisons test. **P <* *0.01.

One possible explanation for the reduced ILC1 frequency and number following *Pc*AS infection could be increased apoptosis. To test this, we stained liver ILC1s *ex vivo* to assess Caspase‐3/7 expression as a marker of apoptosis from days 1 to 4 p.i. (Figure 3a). Flow cytometry analysis revealed approximately 20% of liver ILC1s expressing Caspase‐3/7 in naïve C57BL/6 mice (Figure 3b). Following *Pc*AS infection, the frequency of Caspase‐3/7‐expressing ILC1s increased further at 2 days p.i., compared to naïve cells. Therefore, increased apoptosis may at least partly explain the reduced liver ILC1 frequency early after PcAS infection.

### Emergence of a CD49a^+^ DX5^+^ double‐positive population

**Figure 4:**
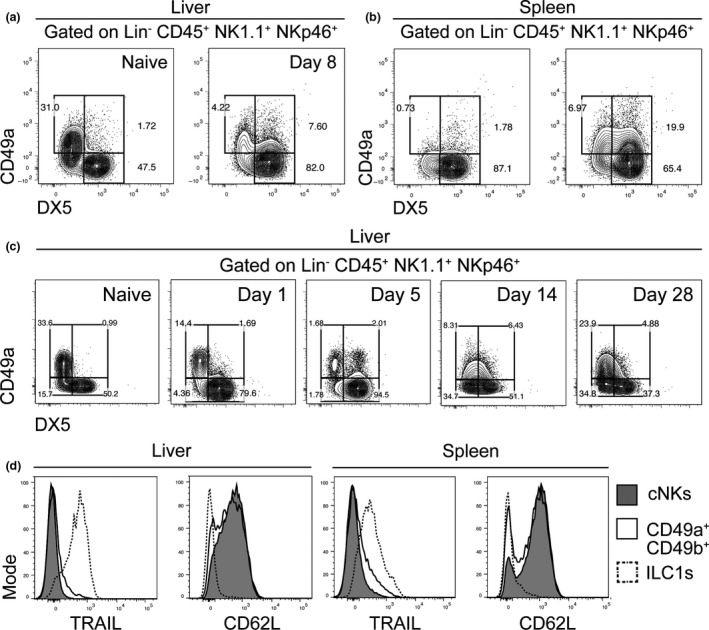
A novel CD49a^+^ DX5^+^ population emerges. Representative plots show a novel CD49a^+^ DX5^+^ double‐positive population in the liver **(a)** and spleen **(b)** of infected mice. The double‐positive population underwent changes during infection **(c)**. Expression of TNF‐related apoptosis‐inducing ligand (TRAIL) and CD62L by the CD49a^+^ DX5^+^ double‐positive population is shown **(d)**. Data are representative of three experiments from cohorts of at least *n* = 4 mice.

We also identified a population of cells within the liver and spleen that were Lin^−^ CD45^+^ NK1.1^+^ NKp46^+^ CD49a^+^ DX5^+^ (herein referred to as the CD49a^+^ DX5^+^ double‐positive' population) (Figures 4a and b). This population was readily detected 5 days p.i. and increased as infection progressed (Figure 4c). This CD49a^+^ DX5^+^ double‐positive population was detected at lower frequencies at 28 days p.i. when the ratio of ILC1s to cNK cells resembled that of naïve samples, although full recovery of ILC1 number or frequency was not evident at this time point (Figure 4c). Interestingly, the CD49a^+^ DX5^+^ double‐positive population expressed the cNK cell marker CD62L and the ILC1 marker TNF‐related apoptosis‐inducing ligand (TRAIL) at intermediate levels (Figure 4d), suggesting they may represent a transitionary population between cNK cells and ILC1s.

### Systemic cNK cell and ILC1 depletion do not affect blood parasitaemia

**Figure 5:**
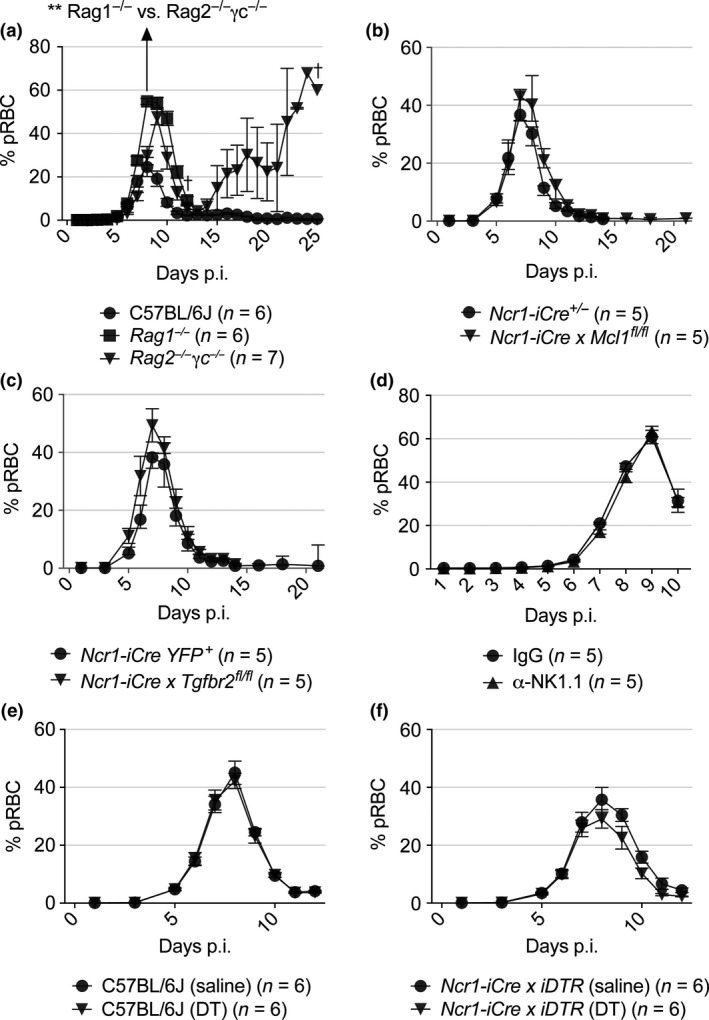
Depletion of cNK cells and ILC1s does not affect parasitaemia. C57BL/6J, *Rag1* ^−*/*−^ and *Rag2* ^−*/*−^ *γ*_*c*_^−*/*−^ mice were infected with *Pc*AS, and the kinetics of infection was measured **(a)**. The kinetics of *Pc*AS infection of *Ncr1‐icre x Mcl1* ^*fl/fl*^ **(b)** and *Ncr1‐icre x Tgfbr2* ^*fl/fl*^ **(c)** mice was compared to that of *Ncr1‐icre* and *Ncr1‐icre* YFP^+^ control mice, respectively. The kinetics of *Pc*AS infection was also measured after administration of the monoclonal antibody (mAb) towards NK1.1 (α‐NK1.1; clone: PK136) **(d)**. Kinetics of parasitaemia in *Ncr1‐icre x iDTR* mice administered with diphtheria toxin is shown alongside the kinetics of parasitaemia for C57BL/6J control mice **(e)**. Data in **a** represent results from three independent experiments with the peak parasitaemia of *Rag1* ^−*/*−^ *,* and *Rag2* ^−*/*−^ *γ*_*c*_^−*/*−^ mice compared using the Mann–Whitney unpaired (nonparametric) *t*‐test. Data in **b, c, d** represent results from one experiment. Error bars represent mean ± SEM. **P <* *0.01.

We next investigated the role of ILC1s during *Plasmodium* infection, given their transcriptional and functional resemblance to Th1 cells,^1^, ^6^ and previous reports indicating important roles for NK cells during *Pc*AS infection.^25^ WT, *Rag1* ^*−/−*^ and *Rag2* ^*−/−*^ *γ*_*c*_^*−/−*^ mice were infected with *Pc*AS, which caused a nonlethal, chronic infection in control WT mice.^33^ Unexpectedly, immunodeficient *Rag2* ^*−/−*^ *γ*_*c*_^*−/−*^ mice (deficient in all lymphocytes) had a delayed peak parasitaemia, compared to *Rag1* ^*−/−*^ mice that were only deficient in B and T cells (Figure 5a). To determine whether the delayed peak parasitaemia observed in *Rag2* ^*−/−*^ *γ*_*c*_^*−/−*^ mice could be attributed to the absence of cNKs, we infected *Ncr1‐iCre x Mcl1* ^*fl/fl*^ mice with *Pc*AS. Myeloid cell leukaemia sequence‐1 (Mcl1) is critical for the maintenance of mature NK cells and ILC1s.^42^ Therefore, these cells were absent in mice lacking *Mcl* gene expression in NKp46 (encoded by the *Ncr1* gene)‐positive cells. Surprisingly, these mice were able to control parasite growth and had similar blood parasitaemia to *Ncr1‐iCre* ^*+/−*^ control mice (Figure 5b). Hence, the delay in peak parasitaemia in *Rag2* ^*−/−*^ *γ*_*c*_^*−/−*^ mice, relative to *Rag1* ^*−/−*^ mice, was not likely caused by the absence of NK cells or ILC1s but instead, possibly reflects changes in either the activity of phagocytic cells or alterations to parasite growth in the blood of *Rag2* ^*−/−*^ *γ*_*c*_^*−/−*^ mice.

We next focused attention specifically on ILC1s by infecting *Ncr1‐iCre x Tgfbr2* ^*fl/fl*^ mice with *Pc*AS. These mice lack *Tgfbr2* gene expression in NKp46‐positive cells and have significantly reduced ILC1 numbers.^6^ Despite this, these mice were still able to control parasite growth as efficiently as control animals (Figure 5c). Given previous contradictory reports on the role of cNK cells during malaria,^25^, ^26^, ^27^, ^28^, ^29^ we next confirmed the above results in 2 other cNK cell‐ and ILC1‐deficient models. First, we treated mice with α‐NK1.1 mAb (depletes cNK cell and ILC1s) and found no effect on the ability to control parasite growth, compared with control mice (Figure 5d). Second, we used diphtheria toxin (DT) to deplete cNK cells and ILC1s in *Ncr1‐iCre x iDTR* mice and again found no change in the ability of mice to control infection, relative to control‐treated animals (Figure 5e). Together, these data indicate limited roles for cNK cells and ILC1s in antiparasitic immunity during *Pc*AS infection.

## Discussion

Here, we show a reduction in the frequency of circulating ILC1s, NK cells and innate‐like T cells in healthy volunteers infected for the first time with *Pf*. This finding was akin to the decrease in frequencies and numbers of peripheral T cells during acute P*f* malaria in Ghanaian children.^43^ Similar observations were made for spleen and liver ILC1s in C57BL/6 mice infected with *Pc*AS, accompanied by increased apoptosis in the latter cell population. Further studies on the roles of ILC1s and NK cells during *Pc*AS infection using cell depletion and genetically modified mice indicated a limited role for these cells in the control of blood parasitaemia. These results contrast earlier findings that reported NK cells confer protection during *Plasmodium* infection using anti‐asialo GM1 to deplete NK cells,^25^ but were consistent with more recent findings using anti‐NK1.1 mAb for NK cell depletion.^28^ Interestingly, a recent study in humans who had received a bone marrow transplant found that ILCs were redundant in the presence of competent B and T cells.^5^ Our data indicate this may also be the case in experimental malaria, but we cannot yet make definitive conclusions on the role of these cells in human malaria.

A recent study has reported that ILCs were irreversibly lost during acute HIV‐1 infection.^44^ Our findings in *Pc*AS‐infected mice indicate that liver and splenic ILC1s were lost in the first 5 days of infection but recovered as infection was controlled. Similarly, in volunteers participating in CHMI studies, the frequency and number of circulating ILC1s, NK cells and innate‐like T cells all fell in the first 7 days of *Pf* infection but recovered after drug treatment. This recovery may have been driven, at least in part, by increased levels of parasite molecules being available after drug‐mediated killing to promote activation and/or expansion of certain immune cell subsets. Nevertheless, changes in these cell populations during malaria were transient, possibly reflecting the less persistent nature of *Plasmodium* infection, compared to HIV. Of note, we cannot exclude the possibility that reduced levels of circulating ILC1s, NK cells, and innate‐like T cells following *Pf* infection or ILC1s following *Pc*AS infection might reflect infection‐mediated changes in the marker expression used to define our cell populations and/or sequestration of cells in tissues. This will require further examination.

We identified a cell population in the mouse liver that expressed both CD49a (an ILC1 marker) and DX5 (a cNK cell marker). This population was also NK1.1‐ and NKp46‐positive and expressed intermediate levels of TRAIL (expressed by ILC1s but not cNK cells) and CD62L (expressed by cNK cells but not ILC1s). These CD49a^+^ DX5^+^ double‐positive cells emerged at 5 days p.i., which coincided with the loss of liver ILC1s. They were also increased in frequency at 14 days p.i., when liver ILC1 frequency had yet to recover but were reduced at 28 days p.i., when the relative proportion of liver ILC1s to cNK cells resembled proportions prior to the loss of liver ILC1s. These cells were not liver‐specific, as they were also found in the spleen. A recent report proposed a bidirectional plasticity between cNKs and ILC1s that was mediated by the presence or absence of Eomesodermin (Eomes).^45^ Our current findings suggest that this CD49a^+^ DX5^+^ double‐positive cell population may be an intermediate population between cNK cells and ILC1s. Recently, we reported a similar cell population expressing CD49a, CD49b and Eomes in mouse tumour models, and this was found to represent an intermediate population between TGFβ‐mediated conversion of cNK cells into ILC1s.^46^ Whether this plasticity between cNKs and ILC1s also occurs in malaria remains unknown but perturbation of TGFβ signalling following infection could help explain the disparity between ILC1 and NK cell frequencies after *Pc*AS infection.

Although our results from mouse models of malaria indicate a limited role for cNK cells and ILC1s in control of parasite growth, we cannot exclude a role for these cells in human malaria, based on our data. Previous studies have shown that human NK cells rapidly produce IFNγ following exposure to *Pf* parasitised red blood cells (pRBCs) *in vitro*.^29^, ^47^ Furthermore, depletion of NK cells in humanised mice infected with *Pf* using anti‐CD56 mAb resulted in increased parasite growth,^48^ albeit parasite growth appeared relatively modest in this model system. Nevertheless, our investigation of associations between cNK cells, ILC and innate‐like T‐cell subsets and either parasite burden (AUC) or PMR revealed no relationships. Further research is clearly needed in this area.

Our CHMI and mouse studies were initiated via an intravenous infusion of blood stage parasites, thereby bypassing the liver stage of infection. One advantage of this is that infections are relatively synchronous and hence, early changes in immune cell populations can be readily detected. However, it is possible that host responses to liver stage infections may influence subsequent responses to blood stage infections.^49^ Recently, a study of innate‐like T cells in CHMI studies in Tanzania using *Pf* sporozoites to establish infection reported changes in the frequencies of circulating NK and MAIT cells up to 6 months after infection, although reduced frequencies of MAIT and NK cells were observed before this time point.^32^ The earliest samples were taken 9 days after infection in this study, making comparisons with our data difficult. Furthermore, the initiation of infection via liver stage, different parasite dose used to establish blood stage infection and the immune status of the Tanzanian volunteers living in a malaria‐endemic region make direct comparisons with our data challenging. Nevertheless, comparisons between CHMI studies initiated with sporozoites and blood stage parasites in healthy volunteers and those living in malaria‐endemic areas are likely to provide a wealth of future data that may help explain the roles of various immune cell populations during infection and disease, especially if infections initiated by liver and blood stages can be directly compared in the same populations.

In conclusion, we report a decline in the frequency of cNK cells, ILC1s, and innate‐like T cells 7 days after blood stage *Pf* infection in CHMI studies with healthy volunteers. A similar observation was made for liver ILC1s in a mouse model of malaria caused by *Pc*AS. Our data indicate a limited role for cNK cells and ILCs in the control of blood parasitaemia in mice. Together, these data provide novel insights into the responses of innate immune cells in mice and humans during malaria and may help guide future strategies aimed at manipulating host immune responses for clinical advantage during this disease.

## Methods

### Controlled human malaria infection

Experimental procedures were performed as part of a substudy on human blood samples collected from consenting participants enrolled in a drug study (Australian New Zealand Clinical Trials Registry ACTRN12613000565741 and NCT02389348) conducted at Q‐Pharm Pty Ltd (Herston, QLD, Australia) under the approval of the QIMR Berghofer Medical Research Institute Human Research Ethics Committee (QIMR‐HREC). Volunteers comprised healthy male and female nonsmokers, aged between 18 and 45 in one trial and 18 and 55 in another trial, with no history of malaria or prior exposure to malaria‐endemic regions. Experiments were performed on blood samples collected from participants enrolled in 3 cohorts (*n* = 14 volunteers in total). An additional cohort (*n* = 8 volunteers) was used to examine NK cell frequency and number. Participants were infected intravenously using a *Pf* (clone 3D7)‐induced blood stage malaria (IBSM) challenge inoculum (1800 parasitised red blood cells [pRBC]), with parasitaemia monitored by real‐time quantitative polymerase chain reaction (qPCR),^50^, ^51^, ^52^ and blood collected at time points indicated in Results section. Antimalarial drug treatment was administered once parasitaemia exceeded 1000 parasites per mL.

Parasitaemia was plotted over time for each patient, and parasite burden over the course of infection (prior to treatment) was expressed as a measurement of AUC, as previously described.^19^ A growth model was also derived from parasitaemia measurements (prior to treatment) over time and fitted to each individual using simple linear regression. The gradient of this growth model (parasite multiplication rate [PMR]) was then estimated using a log‐linear model described by the following equation:

$${\text{log}}_{10}\left(\Upsilon \right)=\alpha +m\times time,$$

where Υ is parasites per mL measured by qPCR, α is the intercept, *m* is the gradient of the growth model, and *time* is the number of days from inoculation.

### Isolation of peripheral blood mononuclear cells from human blood

Approximately 13 mL of blood was collected per volunteer, per time point, in BD Vacutainer^®^ Lithium Heparin^N^ (LH) 170 I.U. Plus Blood Collection Tubes (BD Biosciences, San Jose, CA, USA). Blood was layered over Ficoll‐Paque™ PLUS (GE Healthcare, Little Chalfont, Buckinghamshire, UK) to isolate peripheral blood mononuclear cells (PBMCs). PBMCs were counted using a hemocytometer.

### Mice

Female mice between 8 and 12 weeks old were used. C57BL/6J (WT) mice were purchased from the Walter and Eliza Hall Institute (WEHI), Kew, VIC, Australia. *Rag1* ^*−/−*^, *Rag2* ^*−/−*^ *γ*_*c*_^*−/−*^, *Ncr1‐iCre, Ncr1‐iCre x iDTR, Ncr1‐iCre YFP* ^*+*^, *Ncr1‐iCre x Mcl1* ^*fl/fl*^ and *Ncr1‐iCre x Tgfbr2* ^*fl/fl*^ mice all on the C57BL/6J genetic background were bred in house.^42^, ^53^, ^54^, ^55^, ^56^, ^57^, ^58^, ^59^, ^60^, ^61^ All mice were housed under pathogen‐free conditions at the QIMR Berghofer Medical Research Institute Animal Facility (Herston, QLD, Australia). Experimental use was in accordance with the ‘Australian Code of Practice for the Care and Use of Animals for Scientific Purposes’ (Australian National Health and Medical Research Council) and approved by the QIMR Berghofer Medical Research Institute Animal Ethics Committee (Herston, QLD, Australia; approval number: A02‐633M).

### *Pc*AS infections and measurement of peripheral blood parasitaemia

*Pc*AS was thawed from stabilities and passaged once *in vivo* in a C57BL/6J mouse, prior to establishing experimental infections. Mice were infected intravenously (i.v.) with 10^5^ pRBCs. Parasitaemia was measured by flow cytometry using Hoechst 33342 (Sigma‐Aldrich^®^, St. Louis, MO, USA) and Syto 84 (Invitrogen™, Life Technologies, Carlsbad, CA, USA), as previously described.^62^ Samples were acquired on a BD FACSCanto™ II through BD FACSDiva™ V8.0 (both by BD Biosciences) and analysed on FlowJo v10 OSX (Tree Star, Inc., Ashland, OR, USA).

### Preparation of spleen and liver single‐cell suspensions

Mice were sacrificed by CO_2_ asphyxiation. A mid‐sagittal incision was made on the abdominal cavity. The spleen was removed and passed through a 100‐μm cell strainer (Corning Incorporated, Corning, NY, USA). The single‐cell suspension was washed in Roswell Park Memorial Institute Medium (RPMI; Life Technologies) + 100 μg mL^−1^ penicillin and streptomycin (ps; Gibco^®^, Life Technologies) (RPMI/ps) and incubated for 7 min in Red Blood Cell Lysis Buffer Hybri‐Max™ (Sigma‐Aldrich^®^). Cells were washed in RPMI/ps, pelleted by centrifugation and counted on the Countess II FL (Life Technologies), as per manufacturer's protocol. The liver was perfused with 1× phosphate‐buffered saline (PBS). The excised liver was collected in 1% (v/v) foetal bovine serum (FBS) in PBS and mechanically passed through a 200‐μm square metal mesh. Hepatocytes were separated from lymphocytes and removed using a 33% Percoll™ (GE Healthcare) gradient according to manufacturer's instructions. Red Blood Cell Lysis Buffer Hybri‐Max™ was added to each pellet and incubated for 5 min at room temperature, prior to washing in 1% (v/v) FBS in PBS. Cells were pelleted by centrifugation and counted using the Countess II FL, as per manufacturer's protocol.

### Identification of spleen and liver ILC1s using flow cytometry

Freshly prepared spleen and liver single‐cell suspensions were incubated for 20 min with TruStain fcX™ (anti‐mouse CD16/32; 93) and Zombie Aqua™ Fixable Viability Kit (both from BioLegend, SanDiego, CA, USA) at room temperature. Cells were subsequently stained with a cell lineage cocktail containing biotin‐conjugated anti‐mouse CD3ε (145‐2C11), CD5 (53–7.3) and CD19 (6D5) (all from BioLegend) at room temperature. Lineage‐negative cells in splenocytes were concentrated by negative selection using the EasySep™ Biotin Positive Selection Kit (STEMCELL Technologies™, Vancouver, Canada) according to manufacturer's instructions. Surface staining included fluorophore‐conjugated streptavidin (BioLegend) and fluorophore‐conjugated anti‐mouse monoclonal antibodies (mAb) against CD45 (30‐F11), NK1.1 (PK136), NKp46 (29A1.4), CD49a (HMα1), CD49b (DX5), CD62L (MEL‐14) (all from BioLegend) and TRAIL (CD253, N2B2) (eBioscience, San Diego, CA, USA).

### Identification of human circulatory ILC1s in PBMCs

Freshly isolated PBMCs (2 × 10^7^) were incubated for 3 h with Phorbol 12‐myristate 13‐acetate (PMA) and ionomycin calcium salt (both from Sigma‐Aldrich^®^) in complete media (RPMI/ps + 10% (v/v) FBS) with BD GolgiPlug™ Protein Transport Inhibitor and BD GolgiStop™ Protein Transport Inhibitor (containing Monensin) (both by BD Biosciences) at 37°C. PBMCs were then incubated with a cell lineage cocktail containing fluorescein isothiocyanate (FITC)‐conjugated mouse anti‐human mAbs against CD123 (6H6), TCRα/β (IP26), TCRγ/δ (B1), CD14 (HCD14), CD34 (561), CD19 (HIB19) (BioLegend). PBMCs were incubated with a goat anti‐mouse IgG biotinylated affinity‐purified polyclonal antibody (R&D Systems™ by Bio‐Techne, Minneapolis, Minnesota, USA). Lineage‐negative (Lin^*−*^) cells were isolated using the EasySep™ Biotin Positive Selection Kit (STEMCELL Technologies™) according to manufacturer's instructions.

Lineage‐negative cells were stained with a surface cocktail containing fluorophore‐conjugated anti‐human mAbs towards: CD56 (NCAM; HCD56), CD25 (BC96), Zombie Aqua™ Fixable Viability Kit, CD69 (FN50), and CD127 (A019D5) (all from BioLegend). Cells were then incubated with the Foxp3/Transcription Factor Staining Buffer Set (eBioscience) reagents according to manufacturer's instructions, followed by intracellular staining with an antibody cocktail containing fluorophore‐conjugated anti‐human mAbs against T‐bet (4B10) (eBioscience) and IFNγ (4S.B3) (BioLegend).

### Identification of human innate‐like T cells in PBMCs

PBMCs (5 × 10^5^) were incubated for 3 h with PMA and ionomycin calcium salt in complete media with BD GolgiPlug™ Protein Transport Inhibitor and BD GolgiStop™ Protein Transport Inhibitor (containing Monensin) at 37°C. PBMCs were stained with a surface cocktail containing fluorophore‐conjugated anti‐human mAbs against CD3ε (UCHT1) (both from BD Pharmingen™, BD Biosciences), CD127 (A019D5), CD19 (HIB19) and CD56 (HCD56) (all from BioLegend) or a separate cocktail containing fluorophore‐conjugated anti‐human mAbs against CD161 (DX12) and CD3ε (UCHT1) (both from BD Pharmingen™, BD Biosciences), CD4 (RPA‐T4), CD8α (SK1), Zombie Aqua™ Fixable Viability Kit, TCR Vα7.2 (3C10), TCRγ/δ (B1) (all from BioLegend) and human α‐GalCer‐loaded‐CD1d PBS44 tetramer. In brief, sequences encoding human β2M and the extracellular domain of human CD1d were cloned into the expression vector pHLsec and transfected into mammalian HEK‐293S.GnTI cells.^63^ Human β2M encoding amino acid IQRTP to RDMGS and human CD1d encoding amino acid VPQRL to VLYWGS with a c‐terminal BirA tag and His tag (amino sequence GLNDIFEAQKIEWHEHHHHHH) were purified using Nickel agarose purification and biotinylated using BirA enzyme. Biotinylated CD1d was loaded with PBS‐44 provided by Paul Savage (Brigham Young University, Provo, UT) and converted to tetramers by the addition of streptavidin BV421 (eBioscience).

Cells were incubated with the Foxp3/Transcription Factor Staining Buffer Set (eBioscience) reagents according to manufacturer's instructions, followed by intracellular staining with an antibody cocktail containing fluorophore‐conjugated anti‐human mAbs towards PLZF (R17‐809) and/or IFNγ (4S.B3) (both from BD Pharmingen™).

### Flow cytometry

Samples were resuspended in 1% (w/v) paraformaldehyde (PFA) poststaining and stored at 4°C before acquisition on a BD LSRFortessa™ (special order research product; BD Biosciences) through BD FACSDiva™ V8.0 and analysed on FlowJo v10 OSX.

### Detection of apoptotic cells

Apoptotic cells were detected by cell surface staining with either Annexin V from the Annexin V FITC Apoptosis Detection Kit I (BD Biosciences) or reagents from the CellEvent^®^ Caspase‐3/7 Green Flow Cytometry Assay Kit (Molecular Probes^®^, Life Technologies), as per manufacturer's instructions.

### Depletion of cNKs and ILC1s

C57BL/6J or *Rag1* ^*−/−*^ mice were given an intraperitoneal (i.p.) injection with 1 mg per mouse of either anti‐NK1.1 (PK136) mAb or *InVivo* MAb Polyclonal Mouse IgG (both from BioXCell, West Lebanon, New Hampshire, USA) on alternate days, starting from the day before infection.

*Ncr1‐iCre x iDTR* mice were given 8 ng g^*−*1^ (based on body weight) of DT from *Corynebacterium diphtheria* (Sigma‐Aldrich^®^) per mouse 2 days and 1 day prior to infection and every 2 days after infection. Control mice were given an equal volume of sodium chloride (0.9% [w/v]) for irrigation (Baxter International, Deerfield, IL, USA). Depletion efficacy in both models was determined by flow cytometry analysis using an anti‐mouse mAb towards NKp46 (CD335; 29A1.4) (BioLegend).

### Statistics

Graphing and statistical analyses were performed on GraphPad Prism 6 (GraphPad, San Diego, CA, USA). A *P*‐value (*P*) < 0.05 was considered statistically significant. Direct comparisons between two time points in the human trial data were made using the Wilcoxon (paired, nonparametric) test. For studies involving mice, comparisons between time points in time course experiments were made using the Kruskal–Wallis test accompanied by the Dunn's multiple comparisons test, while comparisons between two groups of mice were made using the Mann–Whitney (unpaired, nonparametric) *t*‐test. All data are shown as mean ± standard error of mean (SEM) unless otherwise stated.
