# Supplementary figures and images for: Rapid loss of group 1 innate lymphoid cells during blood stage Plasmodium infection
Source: Clin Transl Immunology. 2018 Jan 12;7(1):e1003. doi: 10.1002/cti2.1003 (PMC5822408; doi:10.1002/cti2.1003)

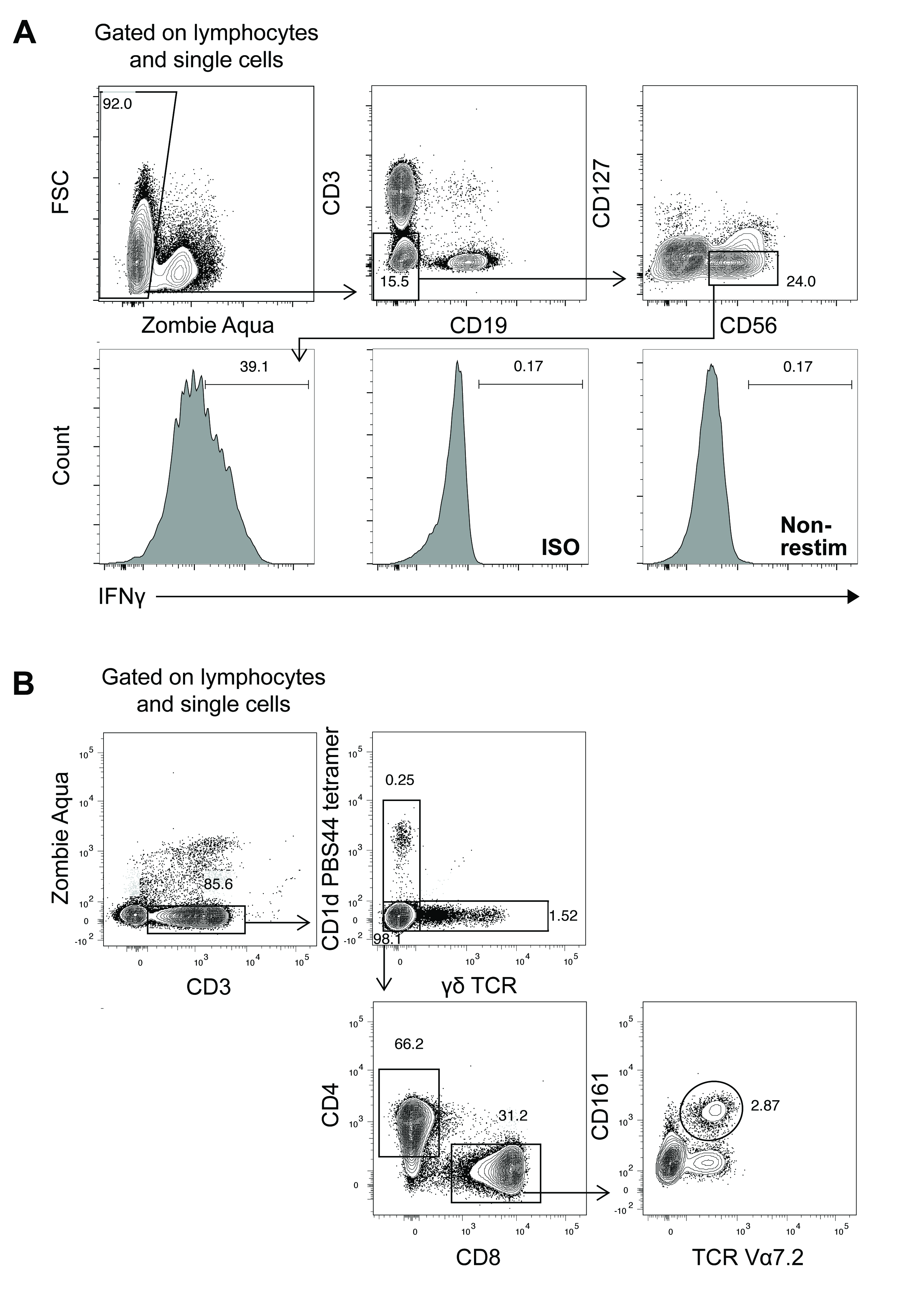

Supplement: Supplementary file 1 [file CTI2-7-e1003-s001.tif]
